# Unsupervised discovery of public health subgroups associated with cognitive health vulnerability in adults: an analysis of BRFSS 2024

**DOI:** 10.3389/fpubh.2026.1878119

**Published:** 2026-09-14

**Authors:** Ziyue Wang, Joshi Dookhy, Hossein Khaleghy, Soumyajyoti Maji, Xuejia Yang, Carmel Geoghegan, Jia Chen

**Affiliations:** 1 School of Nursing, Midwifery & Evidence Science, University of Galway, Galway, Ireland; 2 School of Nursing & Midwifery, University College Dublin, Dublin, Ireland; 3 Galway University Hospitals, Galway, Ireland; 4 School of Computer Science, University of Galway, Galway, Ireland; 5 Discipline of Electrical & Electronic Engineering, University of Galway, Galway, Ireland; 6 Institute for Clinical Trials, University of Galway, Galway, Ireland; 7 Xiangya School of Nursing, Central South University, Changsha, China; 8 Dementia Ireland Empowering Communities, Galway, Ireland

**Keywords:** BRFSS, cognitive health vulnerability, population stratification, public health, subjective cognitive decline, unsupervised representation learning

## Abstract

**Background:**

Cognitive health-related vulnerability is closely associated with multidimensional public health factors, including chronic disease burden, functional limitation, healthcare access, socioeconomic vulnerability, health behaviors, and preventive service utilization. However, latent population heterogeneity embedded in routine public health survey variables remains insufficiently explored.

**Objective:**

This study aims to develop an unsupervised semantic stratification framework based on BRFSS 2024 data to identify adult population subgroups associated with cognitive health-related vulnerability from a public health perspective.

**Methods:**

Routine public health variables were selected, cleaned, imputed, and standardized, while cognitive reference variables were excluded from model training. An autoencoder was used to learn low-dimensional latent representations, followed by MiniBatchKMeans clustering to identify semantic subgroups. Each subgroup was further analyzed through public health profiling, held-out cognitive reference validation, non-redundant feature signature analysis, and intervention priority assessment.

**Results:**

Four interpretable semantic subgroups were identified: the low-burden subgroup, general health vulnerability subgroup, health behavior risk subgroup, and preventive service gap subgroup. These subgroups showed distinct patterns in public health burden, cognitive reference variables, key feature signatures, and intervention priorities.

**Conclusion:**

Routine public health survey data can support the identification of latent adult subgroups associated with cognitive health-related vulnerability and provide exploratory evidence for population monitoring, priority screening, resource allocation, and stratified intervention design. This framework is not a diagnostic, prognostic, or causal inference model, and further validation using weighted, longitudinal, cross-year, and external data is needed.

## 1 Introduction

Subjective cognitive decline has become an important public health concern because it reflects self-reported cognitive difficulties that may affect daily functioning, health service use, caregiving needs, and long-term cognitive health monitoring among adults. Although dementia and related cognitive disorders continue to impose substantial pressure on families, health systems, and social support structures (1, 2), the present study does not aim to diagnose dementia or directly predict clinical dementia outcomes. Instead, it focuses on cognitive health vulnerability in population-level surveillance data, where self-reported cognitive concerns can serve as meaningful signals for identifying groups that may require earlier attention, health education, and targeted public health support (3, 4).

A key challenge in studying cognitive health vulnerability is that it is unlikely to be distributed uniformly across the adult population. Adults differ substantially in chronic disease burden, functional limitation, behavioral patterns, mental health status, healthcare access, socioeconomic resources, and social support. These factors may interact with one another and produce distinct vulnerability profiles rather than a single homogeneous risk pattern. Prior studies have shown that social determinants of health, chronic conditions, metabolic risks, and behavioral factors are closely associated with cognitive aging, dementia-related outcomes, and subjective cognitive decline (5–8). However, most existing studies still rely mainly on predefined variable associations, conventional stratified statistics, or single-factor explanations, which may be insufficient for revealing latent subgroup structures within complex public health survey data.

Unsupervised learning provides a useful methodological pathway for addressing this limitation. Compared with supervised prediction models that require predefined labels, unsupervised methods can explore latent population structures directly from multidimensional public health variables. Representation learning methods can transform high-dimensional survey variables into lower-dimensional latent spaces while preserving essential information and reducing redundancy (9). Deep clustering and embedding-based methods further support the discovery of hidden population patterns from complex health data (10–12). Nevertheless, public health survey data differ from hospital records and clinical trajectories because they contain broader social, behavioral, and self-reported health indicators, often with weaker clinical labels and stronger requirements for interpretability. Therefore, subgroup discovery in this context should not only identify latent data structures but also connect them with meaningful public health and cognitive health vulnerability profiles.

To address these gaps, this study proposes an unsupervised analytical framework for semantic subgroup discovery and stratification of cognitive health vulnerability among adults using BRFSS 2024 data. The framework first standardizes and encodes multidimensional public health variables, learns low-dimensional latent representations, and then performs data-driven clustering in the latent space to identify latent adult subgroups. To improve interpretability, the identified clusters are semantically characterized using multiple public health dimensions, including demographic, behavioral, chronic disease, mental health, healthcare access, and social determinant-related features. Cognitive health-related variables are then introduced as held-out reference indicators to examine whether the discovered subgroups correspond to meaningful differences in subjective cognitive concerns and related functional limitations. This study aims to identify interpretable latent public-health subgroups among adults and examine whether these subgroups differ in cognitive health-related reference indicators without relying on clinical diagnostic labels.

## 2 Data and methods

### 2.1 Dataset

This study used data from the 2024 Behavioral Risk Factor Surveillance System (BRFSS), an annual public health survey conducted in the United States that collects information on demographic characteristics, health behaviors, chronic conditions, functional status, healthcare access, and preventive service use through telephone interviews. BRFSS is suitable for population-level public health analysis because it provides large-scale surveillance data with broad variable coverage and includes a cognitive decline module relevant to subjective cognitive decline and its functional consequences. The final analytic dataset used in this study contained 457,670 records after preprocessing. Table 1 summarizes the basic information of the dataset used in this study.

**Table 1:** Summary of the dataset used in this study.

Item	Description
Dataset	Behavioral Risk Factor Surveillance System, BRFSS 2024
Source	Annual public health survey released by the Centers for Disease Control and Prevention
Population	Community dwelling adults in the United States
Survey mode	Telephone interviews via landline and mobile phones
Coverage	49 states, the District of Columbia, Guam, Puerto Rico, and the US Virgin Islands
Sample size	457,670 valid survey records
Variables	301 original survey and derived variables
Content	Demographics, health behaviors, chronic diseases, functional limitations, health care access, and preventive service use
Cognitive content	Includes a cognitive decline module reflecting subjective cognitive decline and its impact on daily activities and social participation
Research relevance	Suitable for cognitive health factor analysis, deep representation learning, and public health semantic stratification

### 2.2 Data preprocessing

Because the original BRFSS 2024 (13) dataset includes both landline and mobile survey pathways, as well as many interview control fields, sampling identifiers, and derived statistical variables, directly using all fields would introduce irrelevant information and increase redundancy in subsequent representation learning. Therefore, this study first conducted task oriented variable selection for cognitive health related public health analysis. Specifically, interview dates, sample identifiers, telephone pathway screening fields, questionnaire version fields, sampling weights, and other process related variables with limited relevance were removed. In addition, topic specific variables with weak relevance to cognitive health analysis in adults were excluded, including cancer screening, gynecological and prostate related items, caregiver modules, and other less relevant sections. After this step, variables more closely related to demographic characteristics, health status, chronic conditions, functional limitations, health behaviors, health care access, and cognitive health were retained. Special missing codes in the retained fields were then uniformly identified and replaced, and common feature variables were further screened according to missingness. Table 2 summarizes the main preprocessing steps and outcomes.

**Table 2:** Summary of the data preprocessing procedure.

Step	Description
Raw data	The BRFSS 2024 dataset contains 457670 survey records and 301 original variables
Irrelevant variable removal	Removed interview process fields, telephone pathway fields, sample identifiers, questionnaire versions, weights, and weakly related topic variables
Task oriented selection	Retained variables related to demographics, health status, chronic conditions, functional limitations, health behaviors, health care access, and cognitive health
Missing code handling	Treated special codes such as 7, 9, 77, 99, 777, 888, and 999 as missing values
High missing feature removal	Removed common feature variables with missing rates above 50%
Cognitive reference retention	Fully retained five cognitive reference variables, namely CIMEMLO1, CDWORRY, CDDISCU1, CDHOUS1, and CDSOCIA1
Missing value imputation	Applied mode imputation to retained common feature variables, while cognitive reference variables were not imputed
Final data structure	Obtained 35 common feature variables and 5 cognitive reference variables, yielding 40 analysis fields with 457670 samples

After variable selection, retained fields were divided into two groups according to their analytic roles. The first group consisted of common public health feature variables used for representation learning and subgroup discovery, while the second group consisted of held-out cognitive reference variables used only for post hoc validation and interpretation. This separation was intended to reduce information leakage by excluding cognitive reference variables from the representation-learning and clustering stages. The final dataset included 35 common feature variables and five cognitive reference variables. Special missing codes were recoded as missing values, common features with high missingness were removed, and the remaining common features were mode-imputed. Because this analysis was designed as an exploratory subgroup-discovery study, BRFSS sampling weights were not incorporated into model training or clustering. Accordingly, identified subgroup proportions should be interpreted as patterns within the analytic sample rather than direct population prevalence estimates.

### 2.3 Method

#### 2.3.1 Deep representation learning for public health features

After data screening and preprocessing, the common public health features of adult respondents are organized into a matrix **X**∈ℝ^*N*×*d*^, where *N* denotes the number of samples and *d* denotes the number of retained common features. Because different variables have different coding ranges and numerical scales, the input features are first standardized to reduce the effect of scale inconsistency on neural network optimization. For the *j*th feature, the standardized value is defined as


$$\begin{array}{l} {\tilde{x}}_{ij}=\frac{x_{ij}-\mu_{j}}{\sigma_{j}+\varepsilon } \end{array}$$ (1)

where *x*_*ij*_ denotes the original value of the *j*th feature for the *i*th sample, μ_*j*_ and σ_*j*_ denote the mean and standard deviation of that feature over all samples, and ε is a small constant used to avoid division by zero. The standardized input matrix is denoted by $\tilde{X}\sim \in {\mathbb{R}}^{N\times d}$. On this basis, an autoencoder is constructed to learn latent structural information from high dimensional public health variables, its model architecture is shown in Figure 1.

**Figure 1:**
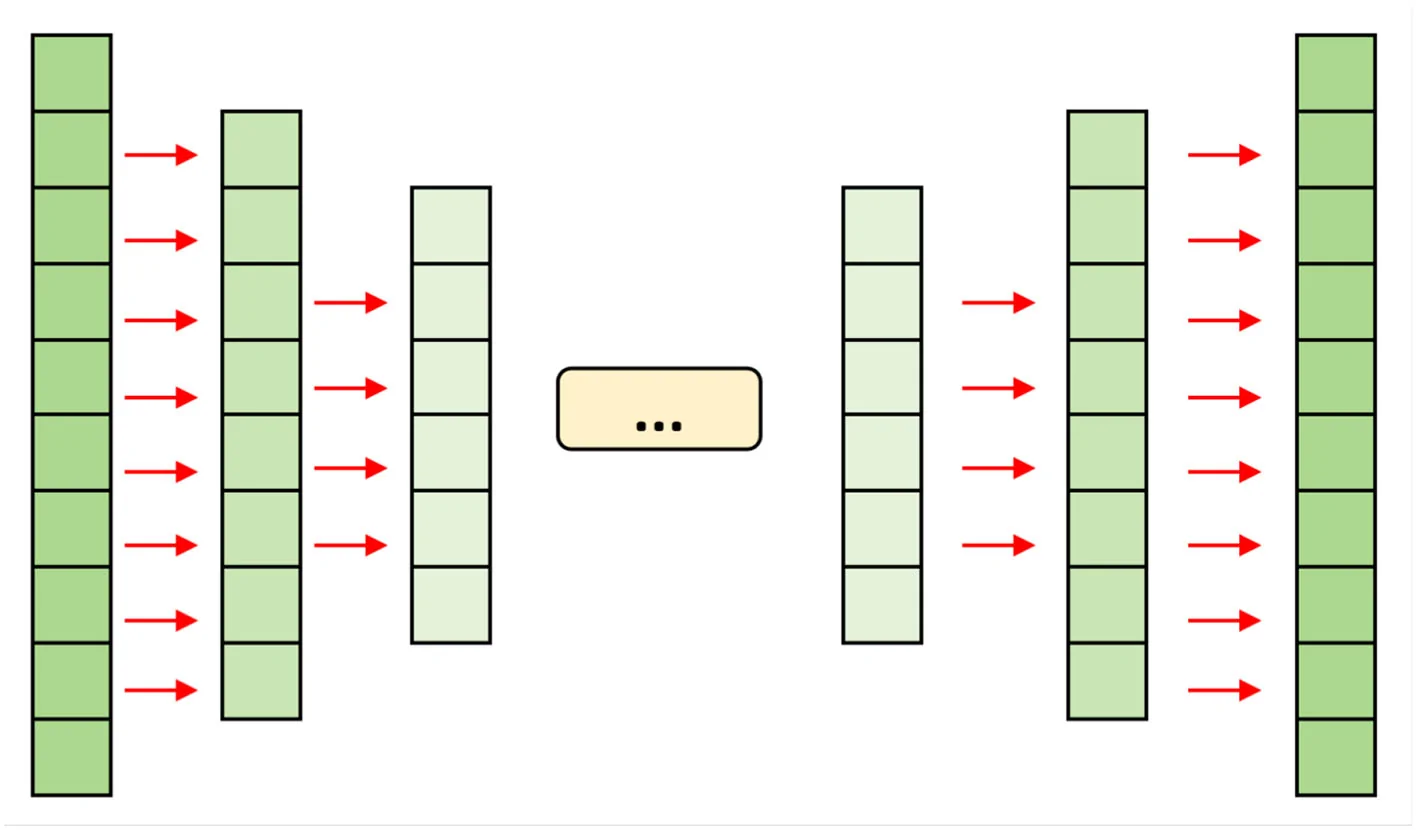
Architecture of the autoencoder used to learn low-dimensional latent representations from high-dimensional public health features.

Let the encoder be denoted by *f*_θ_(·) and the decoder by *g*_ϕ_(·). For sample ${\tilde{X}}_{i}$, the latent representation **z**_*i*_ and the reconstructed output ${\hat{X}}_{i}$ are defined as


$$z_{i}=f_{\theta }\left({\tilde{X}}_{i}\right)$$ (2)

$${\hat{X}}_{i}=g_{\theta }\left(z_{i}\right)$$ (3)

where $z_{i}\in {\mathbb{R}}^{m}$ and *m*≪*d* denote the compressed latent embedding dimension. The encoder and decoder are implemented with multilayer fully connected networks, and nonlinear activations are introduced in hidden layers to model complex interactions among variables. The encoding process at layer *l* is written as


$$h_{i}^{\left(l\right)}=\sigma \left(W_{e}^{\left(l\right)}h_{i}^{\left(l-1\right)}+b_{e}^{\left(l\right)}\right)$$ (4)

where $h_{i}^{\left(0\right)}={\tilde{x}}_{i}$, σ(·) is the nonlinear activation function, and $W_{e}^{\left(l\right)}$ and $b_{e}^{\left(l\right)}$ are the weight matrix and bias vector of the *l*th encoder layer. Similarly, the decoding process is defined as


$${\hat{h}}_{i}^{\left(l\right)}=\sigma \left(W_{e}^{\left(l\right)}{\hat{h}}_{i}^{\left(l-1\right)}+b_{d}^{\left(l\right)}\right)$$ (5)

where ${\hat{h}}_{i}^{\left(0\right)}=z_{i}$ , and the final output is ${\hat{x}}_{i}={\hat{h}}_{i}^{\left(L_{d}\right)}$. Since the goal is not direct supervised classification but the extraction of stable and interpretable latent health representations from common public health features, the reconstruction error is used as the optimization objective. The reconstruction loss over all samples is defined as


$${ℒ}_{rec}=\frac{1}{N}\sum_{i=1}^{N}{\left\|{\tilde{x}}_{i}-{\hat{x}}_{i}\right\|}_{2}^{2}$$ (6)

The model parameters are learned by minimizing $L_{rec}$, that is,


$$\begin{array}{l} \theta^{*},\phi^{*}=\arg\underset{\theta ,\phi }{\min}L_{rec} \end{array}$$ (7)

Through this process, the autoencoder extracts low dimensional latent representations from multi source public health information such as health status, chronic comorbidity, functional limitation, health behaviors, medical access, and socioeconomic attributes without introducing cognitive outcome variables. This representation reduces redundancy in the original feature space and provides a more suitable basis for subsequent semantic subgroup discovery and heterogeneity analysis. After training, the latent vectors produced by the encoder are denoted as $Z={\left[z_{1},z_{2},\ldots ,z_{N}\right]}^{Τ}\in {\mathbb{R}}^{N\times m}$, which serve as the deep representation of public health status for later clustering and semantic interpretation.

#### 2.3.2 Semantic subgroup discovery and external validation with cognitive health

After obtaining the low dimensional latent representations, semantic public health subgroups are identified from the structure of the embedding space. Considering the large sample size, a mini batch clustering strategy is adopted in the latent space. Let {***μ***_1_, ***μ***_2_, …, ***μ***_*K*_} denote the set of cluster centers, where *K* is the predefined number of subgroups. The cluster assignment of the *i*th sample is defined as


$$c_{i}=\arg\underset{k\in \left\{1,\ldots K\right\}}{\min}{\left\|z_{i}-\mu_{k}\right\|}_{2}^{2}$$ (8)

The corresponding clustering objective is


$$J_{clu}=\sum_{i=1}^{N}\sum_{k=1}^{K}r_{ik}{\left\|z_{i}-\mu_{k}\right\|}_{2}^{2}$$ (9)

where *r*_*ik*_∈{0, 1} is an indicator variable that equals 1 when sample **z**_*i*_ is assigned to cluster *k*, and 0 otherwise. During clustering, the center of cluster *k* is updated by the sample mean


$$\mu_{k}=\frac{\sum_{i=1}^{N}r_{ik}z_{i}}{\sum_{i=1}^{N}r_{ik}}$$ (10)

An example of its update is shown in Figure 2.

**Figure 2:**
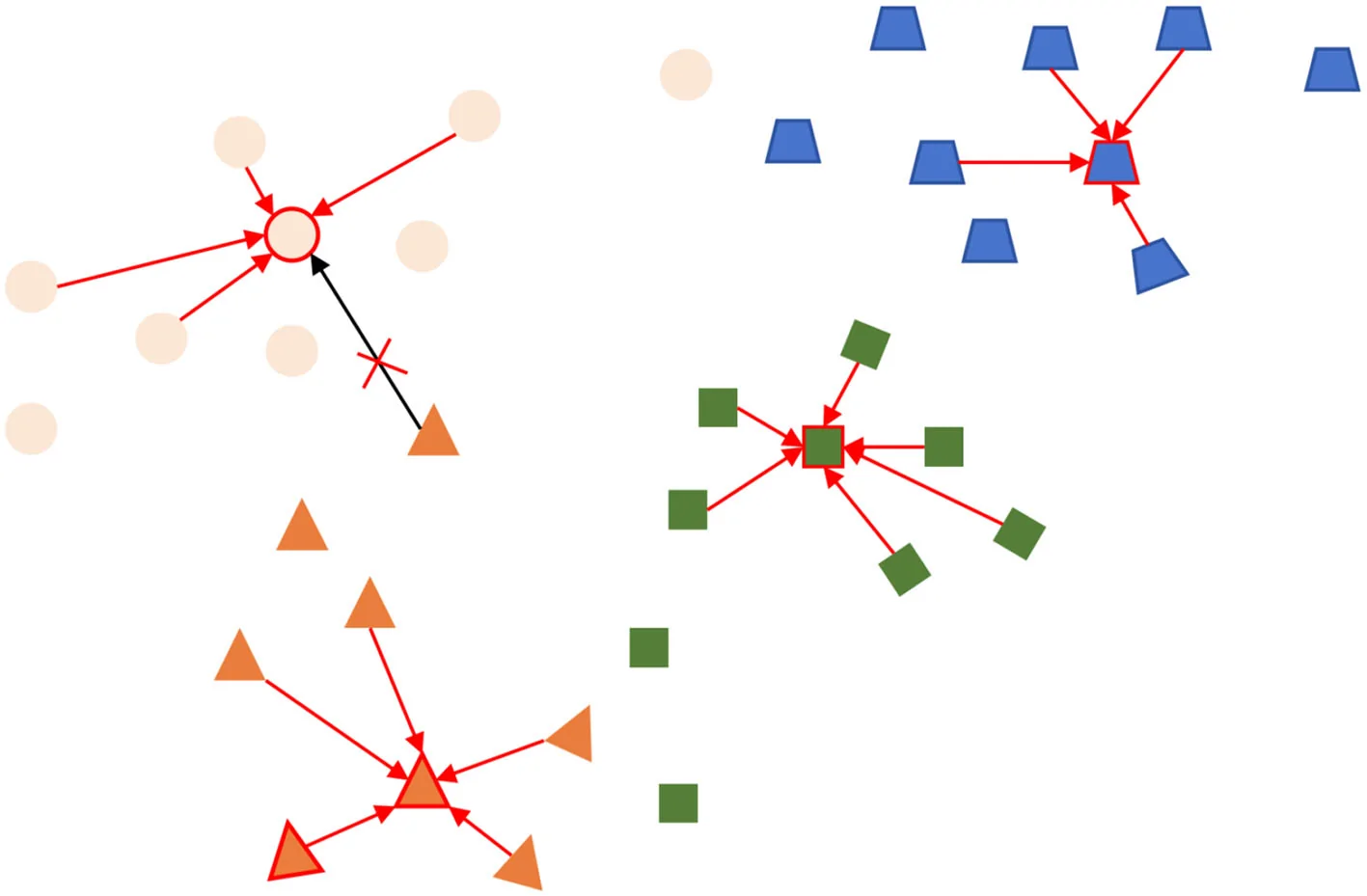
Illustration of cluster center updating in latent space, where each centroid is iteratively adjusted according to the mean position of the samples assigned to the corresponding cluster.

This procedure yields a latent subgroup assignment for each respondent. However, clustering alone only provides formal group labels and does not directly support public health interpretation. Therefore, several task aligned public health dimensions are further constructed, including chronic comorbidity burden, functional limitation, medical access constraint, socioeconomic vulnerability, general health vulnerability, unhealthy behavior, and preventive care gap. The Preventive Care Gap domain was calculated as the mean of two direction-aligned indicators representing non-receipt of influenza and pneumococcal vaccination, with higher values indicating a greater gap in the uptake of these selected preventive vaccination services. Let the *p*th public health dimension be defined by a feature set $S_{p}$. The score of sample *i* on that dimension is defined as


$$\begin{array}{l} d_{ip}=\frac{1}{\left|S_{p}\right|}\underset{j\in S_{p}}{\sum }s_{ij}^{\left(p\right)} \end{array}$$ (11)

where $s_{ij}^{\left(p\right)}$ denotes the direction aligned standardized risk value of feature *j* under dimension *p*. The average profile of cluster *k* on dimension *p* is then defined as


$$\begin{array}{l} {\bar{d}}_{kp}=\frac{1}{N_{k}}\underset{i:c_{i}=k}{\sum }d_{ip} \end{array}$$ (12)

where *N*_*k*_ denotes the number of samples in cluster *k*. The cluster-level average profiles obtained from Equation (12) were subsequently standardized within each public health dimension across clusters to generate the Z-scores used for color encoding in the heatmaps in Figures 3C and 4A; therefore, each Z-score represents the relative burden level of a cluster within a given public health domain. By comparing the average performance of each cluster across different dimensions, each cluster can be mapped to an interpretable semantic label, such as low burden subgroup, chronic comorbidity subgroup, functional limitation subgroup, or socioeconomic disadvantage subgroup. In this way, abstract cluster indices are converted into meaningful public health semantic structures. To further illustrate the distribution pattern of the embedding space, the latent representations are projected into a two dimensional space to visualize the separation and overlap among semantic subgroups.

**Figure 3:**
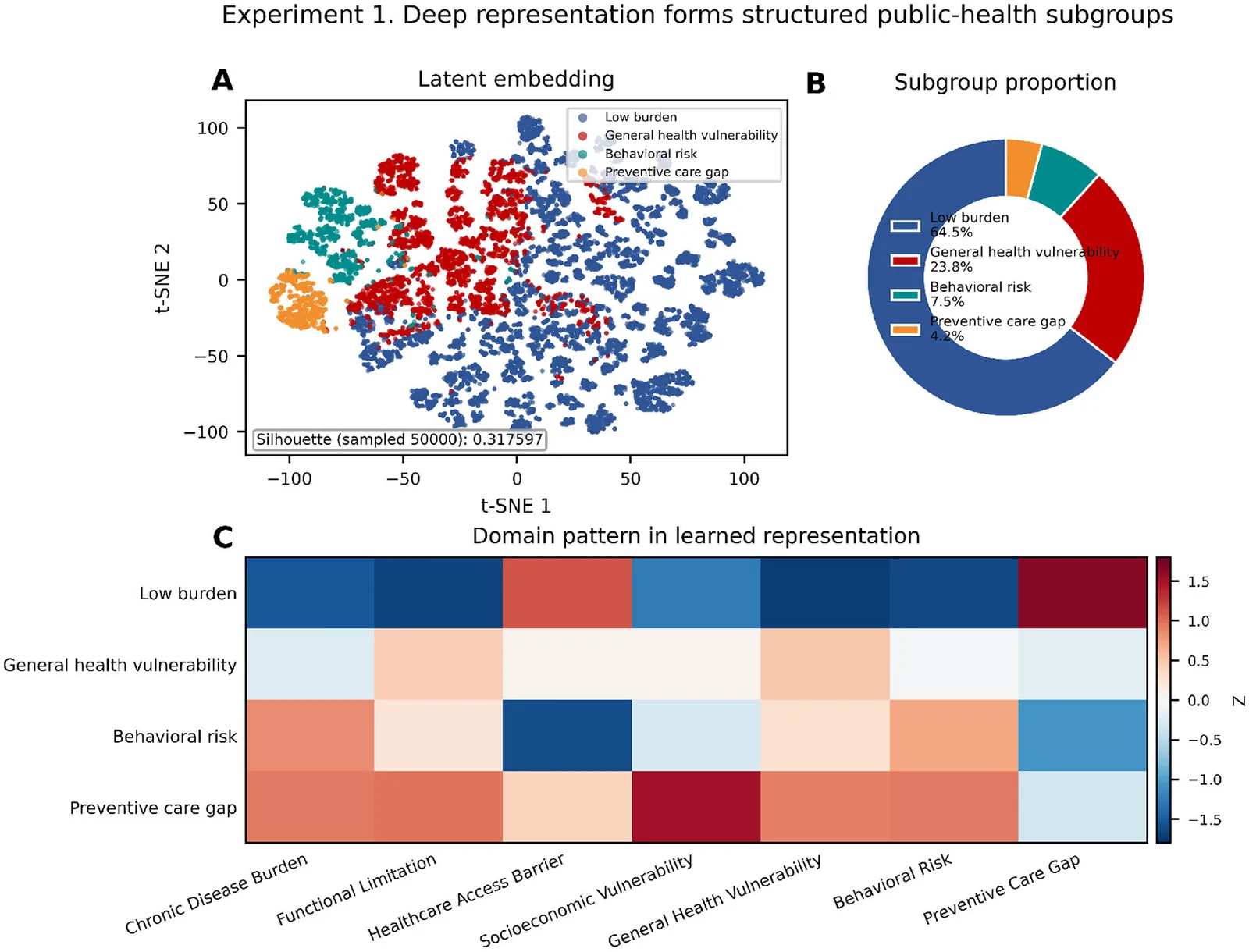
Deep representation learning reveals structured public-health subgroups. **(A)** t-SNE visualization of the latent embedding. **(B)** Proportions of the identified subgroups. **(C)** Domain patterns across the learned subgroups.

**Figure 4:**
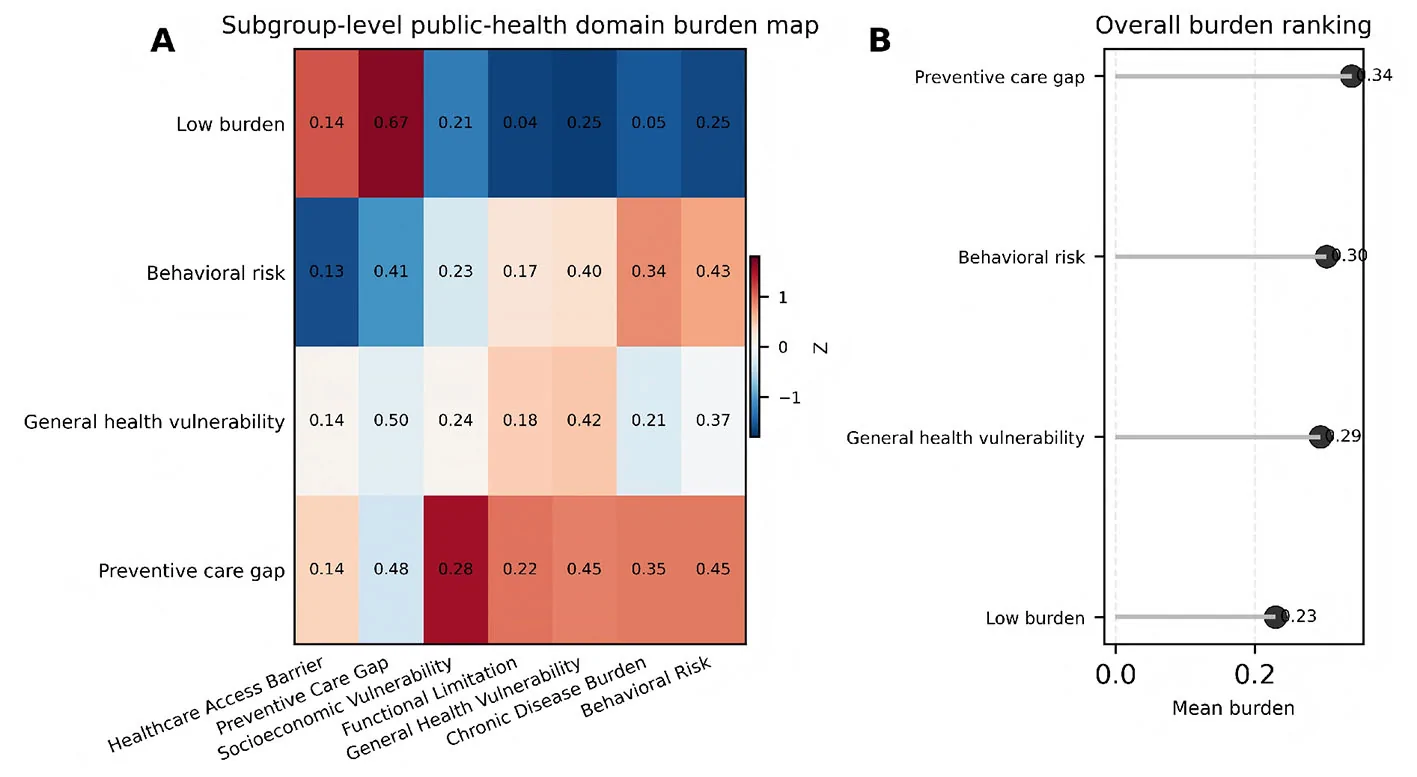
Public-health burden profiles across the identified subgroups. **(A)** Subgroup-level burden across seven public-health domains. **(B)** Overall mean burden ranking of the four subgroups.

After semantic profiling, cognitive variables are not included in representation learning or clustering, but are used as external reference signals to validate the association between identified semantic subgroups and cognitive health. Let the cognitive reference variable set be $C=\left\{C_{1},C_{2},\ldots ,C_{M}\right\}$. This study examines the distributional differences of subjective cognitive decline, worry about memory problems, discussion of cognitive issues, household activity limitation, and social participation limitation across semantic subgroups. For cluster *k* and response level *v* of cognitive variable *C*_*m*_, the conditional proportion is defined as


$$P\left(C_{m}=v|c_{i}=k,R_{im}=1\right)$$

$$\begin{array}{ll} = & \frac{\overset{N}{\underset{i=1}{\sum }}I\left(c_{i}=k\right)I\left(R_{im}=1\right)I\left(C_{im}=v\right)}{\overset{N}{\underset{i=1}{\sum }}I\left(c_{i}=k\right)I\left(R_{im}=1\right)}, \end{array}$$ (13)

where *I*(·) is the indicator function. This proportion describes the structural differences in cognitive health related survey outcomes across public health semantic subgroups. When a subgroup shows a higher proportion of subjective cognitive decline, household function impairment, or restricted social participation, it suggests that the subgroup not only exhibits specific vulnerability in public health dimensions but also shows consistent correspondence with adverse cognitive health outcomes. Based on this design, the overall framework forms a pipeline of deep representation learning of public health features, latent population clustering, semantic dimension mapping, and external cognitive health validation, thereby enabling structured characterization and public health interpretation of cognitive health related heterogeneity in adults without relying on clinical diagnostic labels.

### 2.4 Semantic subgroup number determination and interpretability analysis

To ensure that the latent semantic subgrouping results not only exhibit satisfactory statistical aggregation quality but also form stable group structures with clear public health significance, this study determined the number of subgroups by jointly considering internal clustering metrics and result interpretability. Specifically, after obtaining low-dimensional latent representations from the autoencoder, clustering was conducted under different candidate numbers of clusters, ranging from *K* = 2 to *K* = 8. All candidate solutions used the same MiniBatchKMeans settings and random seed, and clustering metrics were calculated using the same sample of 50,000 respondents to ensure comparability. The clustering quality was evaluated from the two perspectives of intra-cluster compactness and inter-cluster separation. For any sample **z**_*i*_, let *a*_*i*_ denote the average distance between this sample and other samples within the same cluster, and let *b*_*i*_ denote the average distance between this sample and samples in the nearest neighboring cluster. The silhouette coefficient of this sample can then be expressed as


$$\begin{array}{l} s_{i}=\frac{b_{i}-a_{i}}{\max\left(a_{i},b_{i}\right)} \end{array}$$ (14)

Taking the average over all samples yields the overall silhouette coefficient


$$\begin{array}{l} S=\frac{1}{N}\overset{N}{\underset{i=1}{\sum }}s_{i} \end{array}$$ (15)

Meanwhile, to further measure the balance between inter-cluster dispersion and intra-cluster cohesion, the Calinski–Harabasz index was introduced as an auxiliary criterion, which is defined as


$$\begin{array}{l} \text{CH}=\frac{\text{Tr}\left(B_{K}\right)/\left(K-1\right)}{\text{Tr}\left(W_{K}\right)/\left(N-K\right)} \end{array}$$ (16)

where *B*_*K*_ denotes the between-cluster dispersion matrix, *W*_*K*_ denotes the within-cluster dispersion matrix, *K* denotes the number of clusters, and *N* denotes the total number of samples. The Davies–Bouldin index was also calculated as a complementary measure, with a lower value indicating greater within-cluster compactness and stronger separation between clusters. By jointly comparing the silhouette coefficient, Calinski–Harabasz index, and Davies–Bouldin index under different values of *K*, the bias caused by relying on a single metric alone can be avoided, thereby providing a more robust quantitative basis for determining the number of subgroups. The quantitative results for the candidate cluster numbers are summarized in Table 3.

**Table 3:** Internal clustering performance under different numbers of semantic subgroups across five independent random seeds.

Number of clusters (*K*)	Silhouette ↑	CH index ↑	DB index ↓
2	0.228312 ± 0.005699	14354.655935 ± 159.906703	1.523149 ± 0.018170
3	0.230107 ± 0.003886	14269.656996 ± 40.750531	1.529909 ± 0.051586
4	**0.320231 ± 0.005097**	**15101.797169 ± 146.683758**	**1.492168 ± 0.021467**
5	0.173336 ± 0.006442	12764.912357 ± 130.874900	1.740787 ± 0.009608
6	0.165511 ± 0.008416	11734.692943 ± 175.425490	1.720957 ± 0.019505
7	0.167044 ± 0.005108	11039.175703 ± 223.070102	1.570928 ± 0.006415
8	0.183039 ± 0.004065	10768.582116 ± 61.030900	1.565429 ± 0.012898

On this basis, this study further screened candidate clustering schemes by incorporating their public health semantic interpretability. When the number of clusters is too small, populations with substantially heterogeneous risk characteristics may be compressed into the same subgroup, making it difficult to reveal differentiated structures related to chronic disease burden, functional limitation, barriers to healthcare accessibility, socioeconomic vulnerability, and unhealthy behaviors. In contrast, when the number of clusters is too large, the population may be excessively fragmented, causing some subgroups to reflect only local variable fluctuations and thereby weakening the stability of the overall structure and its practical interpretive value. As shown in Table 3, across five independent random seeds, the four-cluster solution achieved the highest mean silhouette score of 0.320231 ± 0.005097 and the highest mean Calinski–Harabasz index of 15101.797169 ± 146.683758, together with the lowest mean Davies–Bouldin index of 1.492168 ± 0.021467 among all candidate solutions. The consistent superiority of these three internal metrics indicates that *K* = 4 provides the most compact and clearly separated clustering structure. Considering both internal clustering performance and semantic profiling results, the four-subgroup solution was found to achieve a relatively appropriate balance between statistical stability and public health interpretability. On the one hand, under this partition, the subgroups showed relatively clear boundaries and good compactness in the latent space. On the other hand, these subgroups could be stably aligned with semantic structures carrying explicit public health meaning, including low burden, general health vulnerability, behavioral risk, and preventive care gap. Therefore, this study ultimately selected *K* = 4 as the number of semantic subgroups to support subsequent analyses of external cognitive health validation, multidimensional burden profiling, and comprehensive risk stratification.

### 2.5 Non-redundant feature signature and intervention-priority analysis

To identify subgroup-specific feature signatures while avoiding repeated assignment of the same feature to multiple subgroups, this study calculated a subgroup-level standardized score for each direction-aligned public health feature. Let ${\bar{x}}_{kj}$ denote the mean transformed burden value of feature *j* among respondents assigned to subgroup *k*, defined as


$$\begin{array}{c} {\bar{x}}_{kj}=\frac{1}{N_{k}}\underset{i:c_{i}=k}{\sum }x_{ij}^{\left(\text{risk}\right)}, \end{array}$$ (17)

where *N*_*k*_ is the number of respondents in subgroup *k*, and $x_{ij}^{\left(\text{risk}\right)}$ denotes the direction-aligned burden value of feature *j* for respondent *i*. To quantify the relative specificity of feature *j* across the identified subgroups, the subgroup-level means were standardized across the *K* subgroups:


$$\begin{array}{c} z_{kj}=\frac{{\bar{x}}_{kj}-\mu_{j}}{s_{j}+\epsilon }, \end{array}$$ (18)

where μ_*j*_ and *s*_*j*_ denote the mean and standard deviation of ${\bar{x}}_{kj}$ across the *K* subgroups, respectively, and ϵ is a small constant used to avoid division by zero. Each feature was assigned exclusively to the subgroup with the largest standardized score:


$$\begin{array}{c} k_{j}^{*}=\arg\underset{k\in \left\{1,\ldots ,K\right\}}{\max}z_{kj}. \end{array}$$ (19)

Accordingly, the non-redundant feature-signature set of subgroup *k* was defined as


$$\begin{array}{c} U_{k}=\left\{j|k_{j}^{*}=k\right\}, \end{array}$$ (20)

and its signature size was calculated as $\left|U_{k}\right|$. This exclusive assignment ensured that each feature contributed to the signature of only one subgroup. The complete subgroup-by-feature standardized score matrix was visualized in Figure 5A, the number of exclusively assigned signature features was reported in Figure 5B, and the eight representative features with the largest values of $\underset{k}{\max}z_{kj}$ were displayed in Figure 5C.

**Figure 5:**
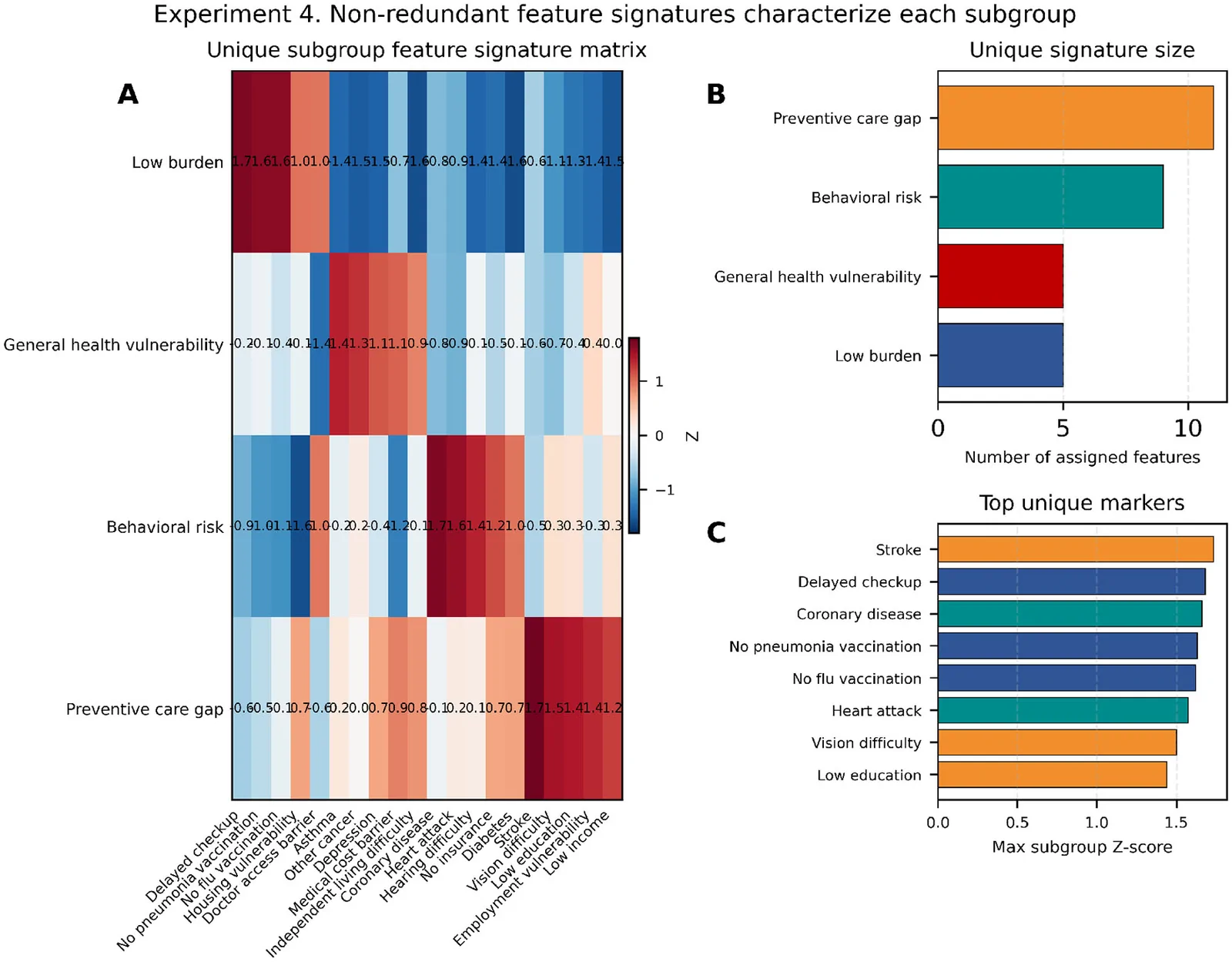
Non-redundant key feature signature analysis results of different semantic subgroups. The figure presents the differentiated public health profiles of adult subgroups from three perspectives: the subgroup feature signature matrix, the number of signature features, and representative key variables.

For the intervention-priority analysis, the overall public health burden of subgroup *k* was calculated as the mean of its seven subgroup-level domain burden scores:


$$\begin{array}{c} B_{k}^{\left(\text{PH}\right)}=\frac{1}{P}\overset{P}{\underset{p=1}{\sum }}{\bar{d}}_{kp},\;P=7, \end{array}$$ (21)

where ${\bar{d}}_{kp}$ is the subgroup-level mean burden score for public health domain *p* obtained from Equation (12). The cognitive-related burden $B_{k}^{\left(\text{Cog}\right)}$ was calculated as the subgroup mean of the respondent-level composite cognitive burden defined in the held-out cognitive reference analysis. In the intervention-priority quadrant map, the horizontal and vertical reference lines corresponded to the mean values of $B_{k}^{\left(\text{PH}\right)}$ and $B_{k}^{\left(\text{Cog}\right)}$ across the four subgroups, respectively, while bubble area was proportional to the subgroup sample size.

To determine subgroup-specific intervention pathways, the seven domain burden scores ${\bar{d}}_{kp}$ were ranked in descending order within each subgroup. The three domains with the highest burden scores were retained as the primary intervention dimensions and were assigned priority ranks of 1, 2, and 3. The subgroup-to-domain pathway diagram linked each subgroup to these three highest-ranked domains. For visualization, the width and opacity of each retained pathway were proportional to its min–max normalized domain burden score:


$$\begin{array}{c} {\tilde{d}}_{kp}=\frac{{\bar{d}}_{kp}-d_{\min}}{d_{\max}-d_{\min}+\epsilon }, \end{array}$$ (22)

where *d*_min_ and *d*_max_ denote the minimum and maximum burden scores among all retained subgroup–domain pathways. The same within-subgroup ranking was used to construct the targeted intervention-signature matrix, in which values of 1, 2, and 3 indicate the first-, second-, and third-priority intervention domains, respectively.

## 3 Experimental results and analysis

### 3.1 Experimental setup

To evaluate the effectiveness of the proposed method for public health semantic profiling related to cognitive health in adults, experiments were conducted on the BRFSS 2024 dataset. In the experiments, the preprocessed common public health features were used to construct the input matrix, while the cognitive reference variables were treated as external validation information rather than training signals in order to avoid information leakage during representation learning. In terms of model implementation, an autoencoder was adopted as the backbone for deep representation learning. The encoder compressed high dimensional public health variables into low dimensional latent representations, and MiniBatchKMeans was then applied in the latent space to identify semantic subgroups among respondents. After that, t-SNE was used to project the latent embeddings into a two dimensional space, and the clustering results were further interpreted and externally validated by combining public health dimension profiles with the distributions of cognitive reference variables. The experiments were implemented with Python, PyTorch, and scikit-learn. Adam was used as the optimizer, mean squared error was used as the reconstruction loss, the number of clusters was set to 4, and clustering performance was evaluated by the sampled silhouette coefficient. To ensure reproducibility, all experiments used a fixed random seed and unified data processing and parameter settings. The main experimental settings are summarized in Table 4.

**Table 4:** Experimental settings.

Parameter	Value
Dataset	Preprocessed BRFSS 2024 data
Common feature dimension	35
Number of cognitive reference variables	5
Number of samples	457670
Deep representation model	AutoEncoder
Input dimension	35
Encoder hidden layers	128, 64
Latent representation dimension	8
Decoder structure	Symmetric to the encoder
Batch size	2048
Training epochs	50
Optimizer	Adam
Learning rate	0.001
Reconstruction loss	Mean Squared Error
Clustering method	MiniBatchKMeans
Number of clusters	4
Clustering batch size	10000
Clustering iterations	20
Embedding visualization	t-SNE
t-SNE sample size	20000
Silhouette coefficient	Sample based computation
Silhouette sample size	50000
Implementation framework	Python, PyTorch, scikit-learn

### 3.2 Experimental results and analysis

#### 3.2.1 Deep representation structure and semantic subgroup overview

To examine whether the low-dimensional representations learned from public health variables can characterize latent structures within the adult population, this section first provides an overall visualization analysis of the deep representation space, semantic subgroup composition, and public health dimensional distributions, as shown in Figure 3. This analysis aims to provide a basis for subsequent public health semantic interpretation, validation using cognitive reference variables, and intervention priority analysis, without interpreting the clustering results as clinical diagnostic categories or deterministic risk stratification.

Figure 3A shows that different semantic subgroups form relatively distinguishable distribution patterns in the latent representation space, indicating that the model does not merely produce random clusters but can learn population representations with a certain degree of internal consistency from adult public health variables. Specifically, the low-burden subgroup occupies a broader region, whereas the subgroups associated with health vulnerability, behavioral risk, and preventive-care gaps are more concentrated in local areas. Figure 3B further shows that the low-burden subgroup accounts for the largest proportion, while the general health vulnerability subgroup also represents a substantial share, suggesting that although most adults are in a relatively low-risk state, a non-negligible subgroup with health vulnerability still exists. Figure 3C explains these subgroup differences from the perspective of public health domain features: the low-burden subgroup exhibits a generally lower risk level, the general health vulnerability subgroup shows stronger feature responses in chronic disease burden, functional limitation, and general health vulnerability, the behavioral risk subgroup is characterized by more prominent behavior-related factors, and the preventive-care gap subgroup shows more evident patterns in healthcare access, socioeconomic vulnerability, and preventive care. Overall, Figures 3A–C jointly demonstrate that the proposed unsupervised representation learning framework can identify latent population heterogeneity structures with clear public health implications from adult public health data without relying on cognitive health labels.

To further quantify whether the learned latent representations improved subgroup separation, MiniBatchKMeans was additionally applied directly to the standardized 35-dimensional common feature space and compared with clustering based on the eight-dimensional autoencoder representations, as summarized in Table 5.

**Table 5:** Comparison of clustering performance between the standardized original feature space and the learned latent representation space across five independent random seeds.

Input representation	Dimension	Silhouette ↑	CH index ↑	DB index ↓
Standardized common features	35	0.044120 ± 0.001187	2835.892313 ± 51.784281	3.190973 ± 0.052752
Learned latent representations	8	**0.320231 ± 0.005097**	**15101.797169 ± 146.683758**	**1.492168 ± 0.021467**

Under the same four-cluster setting and using the same evaluation sample of 50,000 respondents, clustering based on the autoencoder latent representations achieved a substantially higher mean silhouette score than direct clustering on the standardized common features across five independent random seeds, increasing from 0.044120 ± 0.001187 to 0.320231 ± 0.005097. The latent-representation approach also produced a higher mean Calinski–Harabasz index of 15101.797169 ± 146.683758, compared with 2835.892313 ± 51.784281 for the standardized common features, and a lower mean Davies–Bouldin index of 1.492168 ± 0.021467, compared with 3.190973 ± 0.052752. Because higher silhouette and Calinski–Harabasz values and a lower Davies–Bouldin value indicate more compact and better-separated clusters, these consistent results demonstrate that the encoder-derived latent representations provide a more suitable feature space for semantic subgroup discovery than direct clustering on the original standardized features.

#### 3.2.2 Public health semantic profiling of identified subgroups

To further interpret the public health implications of the latent subgroups within the adult population, this section systematically characterizes the identified semantic subgroups across multiple dimensions, including chronic disease burden, functional limitation, barriers to healthcare access, socioeconomic vulnerability, general health vulnerability, health behavior risk, and preventive service gaps. This analysis focuses on the relative differences among subgroups in terms of their public health feature combinations, with the aim of examining whether the population structures formed in the latent representation space carry interpretable public health semantics, as shown in Figure 4.

Figure 4 presents the structured differences among the identified semantic subgroups across public health dimensions. Figure 4A shows that the subgroups differ not merely in a single variable, but in combined patterns across multiple public health domains. In this panel, the color scale represents the domain-wise standardized burden level, whereas the numerical annotation in each cell reports the corresponding raw subgroup-level mean burden score. Specifically, the preventive service gap subgroup exhibits relatively high burdens in socioeconomic vulnerability, general health vulnerability, chronic disease burden, and health behavior risk, whereas the low-burden subgroup shows a generally lower overall burden while still retaining certain characteristics in the preventive service gap dimension. Figure 4B ranks the subgroups according to their mean burden across all public health domains and shows that the preventive service gap, health behavior risk, and general health vulnerability subgroups have relatively higher overall public health burdens, suggesting that these subgroups may represent population structures requiring greater public health attention.

#### 3.2.3 Held-out cognitive reference validation

To further examine whether the adult semantic subgroups derived from public health variables are consistently associated with cognitive health-related information, this section introduces cognitive reference variables that were not involved in representation learning or clustering for supplementary analysis, as shown in Figure 6. It should be emphasized that these variables serve only as independent reference evidence outside the modeling inputs to evaluate the cognitive health relevance of the subgroup partitioning, rather than as clinical diagnostic labels or supervised prediction targets.

**Figure 6:**
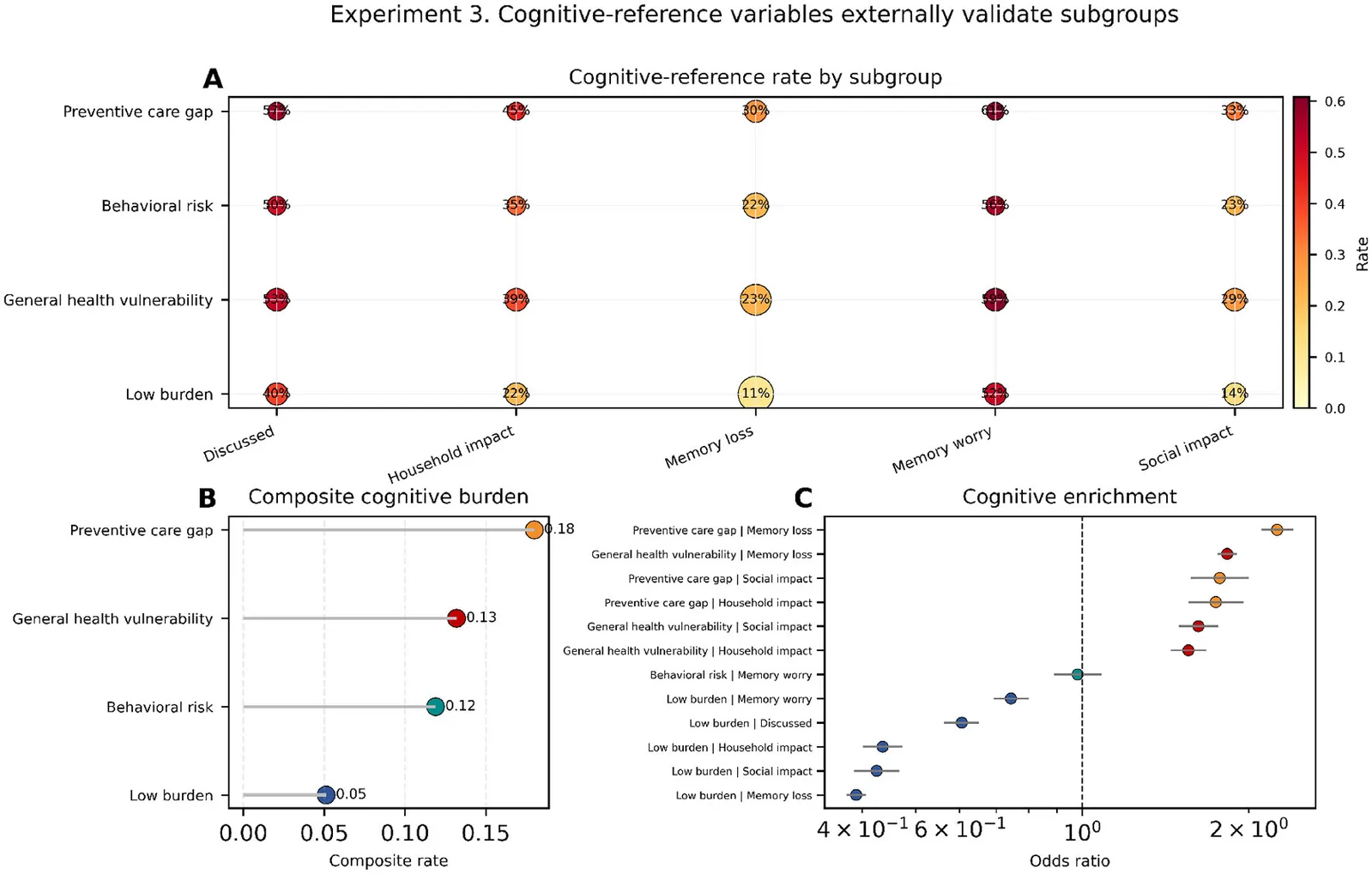
Supplementary validation of adult semantic subgroups using cognitive reference variables that were not involved in representation learning or clustering. The figure shows the associations between different subgroups and cognitive health-related information from three perspectives: the proportions of cognitive reference variables, the composite cognitive-related burden, and the enrichment degree of cognitive variables. **(A)** The numerical labels indicate within-subgroup response rates and bubble sizes indicate the corresponding numbers of valid responses. **(B)** presents the subgroup-level composite cognitive burden, while **(C)** presents odds ratios and 95% confidence intervals comparing each subgroup with all remaining subgroups.

Because the BRFSS Cognitive Decline module was administered only to respondents aged 45 years or older in selected states and questionnaire versions, the cognitive-reference analyses were restricted to respondents with valid responses to the corresponding cognitive variable. The module-available sample was operationally identified as respondents aged at least 45 years who belonged to a state–questionnaire-version stratum in which the first cognitive item, CIMEMLO1, was observed. As shown in Figure 7, the complete analytic sample included 457,670 respondents, of whom 317,564 were aged 45 years or older and 91,804 were included in the operationally identified module-available sample. Valid responses were defined as codes 1 or 2, and variable-specific valid sample sizes were used because the subsequent cognitive items were subject to questionnaire skip patterns. The valid sample sizes were 78,019 for CIMEMLO1, 12,919 for CDWORRY, 12,976 for CDDISCU1, 12,936 for CDHOUS1, and 12,848 for CDSOCIA1.

**Figure 7:**
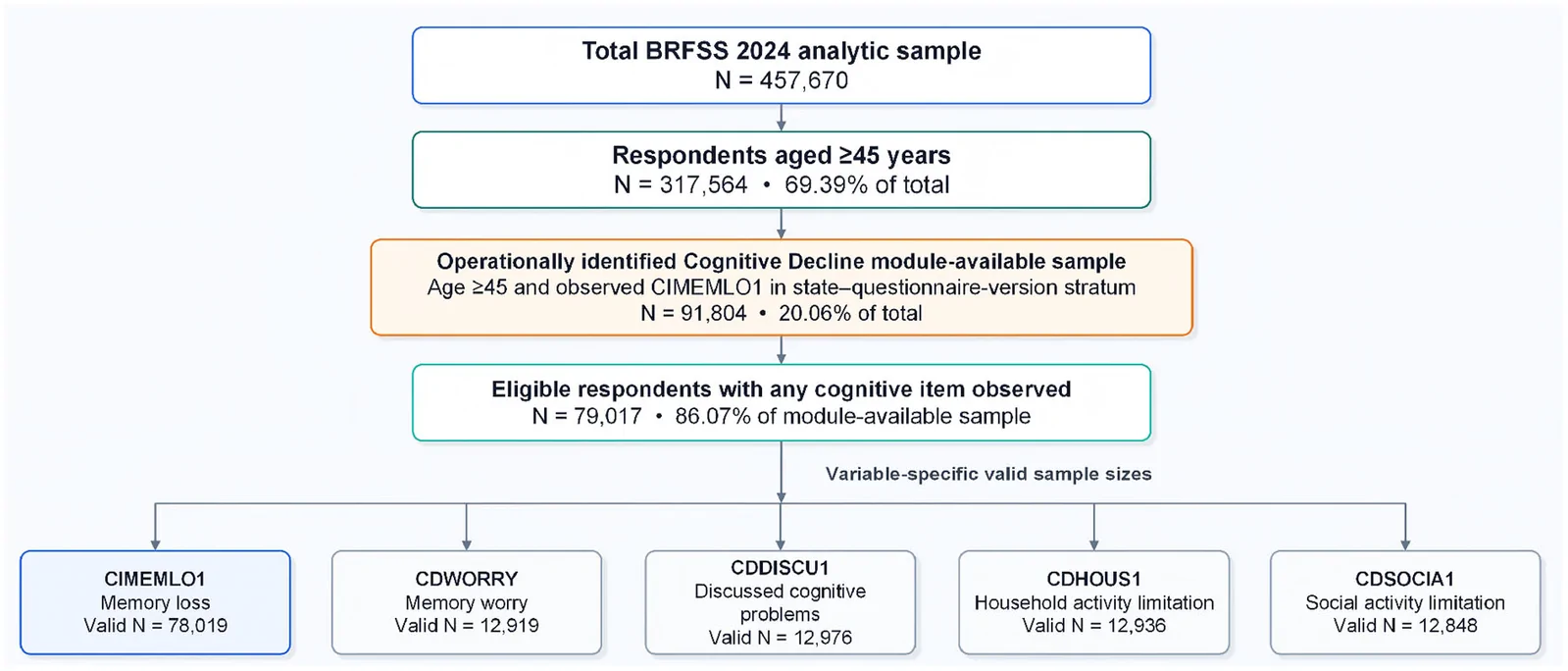
Flowchart of sample selection and variable-specific valid sample sizes for cognitive decline-related measures in the 2024 BRFSS dataset.

In Figure 6A, the percentage shown within each bubble represents the proportion of respondents with a response coded as 1 among those with valid responses to the corresponding cognitive reference variable within each subgroup, whereas the bubble size is proportional to the number of valid responses. In Figure 6B, the composite cognitive burden was calculated using memory loss, memory worry, household activity limitation, and social activity limitation. Each variable was coded as 1 for an adverse response and 0 for a non-adverse response, after which the available indicators were averaged for each respondent and subsequently averaged within each subgroup. In Figure 6C, the odds ratio compares the odds of a response coded as 1 in a given subgroup with the corresponding odds in all remaining subgroups combined. A continuity correction of 0.5 was applied to the four cells of the contingency table, and the 95% confidence interval was calculated on the logarithmic odds-ratio scale.

Figure 6 illustrates the correspondence between the semantic subgroups and the held-out cognitive reference variables. Figure 6A shows that different semantic subgroups exhibit clear differences in cognitive reference variables, including memory loss, memory concerns, limitations in household activities, limitations in social activities, and whether related problems had been discussed, indicating that the subgroup structure learned from public health variables can capture certain cognitive health-related heterogeneity. Compared with the behavioral risk subgroup, the general health vulnerability subgroup showed a higher rate of household activity limitation (49% versus 35%) and social activity limitation (29% versus 23%), while their memory-loss rates were similar (23% versus 22%). Consistently, the composite cognitive burden of the general health vulnerability subgroup was slightly higher than that of the behavioral risk subgroup (0.13 versus 0.12). This pattern may reflect the closer coexistence of poor general health and functional limitations with cognitive-related difficulties in daily activities; however, these descriptive differences should not be interpreted as evidence of a causal relationship. Figure 6B further integrates multiple cognitive reference variables into a composite cognitive-related burden, showing that the preventive service gap subgroup, the general health vulnerability subgroup, and the health behavior risk subgroup have relatively higher composite burdens, whereas the low-burden subgroup remains at a lower overall level. This suggests that subgroups with heavier public health burdens tend to be accompanied by more pronounced cognitive-related problems. Figure 6C characterizes the relative enrichment of specific cognitive reference variables across subgroups in the form of odds ratios, further demonstrating that several high-burden subgroups are more strongly associated with memory concerns, household activity impairment, and social activity impairment, while the low-burden subgroup generally shows a lower level of cognitive-related enrichment.

The distributions of age, state, questionnaire version, and cognitive-variable response and missing-status categories across the four semantic subgroups are reported in the Supplementary Tables.

#### 3.2.4 Non-redundant key feature signature analysis

To further improve the clarity of semantic subgroup interpretation, this section conducts a non-redundant signature analysis of the key public health features for each subgroup within the adult population, thereby avoiding the repeated use of the same variables across multiple subgroup explanations and preserving subgroup distinctiveness. This analysis aims to identify the most representative combinations of public health features for each semantic subgroup, providing clearer variable-level evidence for subsequent population profiling interpretation and public health intervention priority analysis.

Figure 7 presents the non-redundant public health feature signatures of each semantic subgroup. Figure 7A shows that different subgroups exhibit clear differences in their assigned representative features, with the low-burden subgroup, general health vulnerability subgroup, health behavior risk subgroup, and preventive service gap subgroup corresponding to distinct feature combinations. This indicates that these subgroups are not repeatedly explained by the same set of variables, but instead have relatively independent foundations for population profiling. Figure 7B further shows the number of non-redundant signature features associated with each subgroup, revealing that the scale of interpretable features differs across subgroups and suggesting heterogeneity in their public health characteristics. Figure 7C lists the most discriminative key features, among which stroke, delayed routine checkup, coronary heart disease, vaccination gaps, heart attack, vision difficulty, and low educational attainment show relatively high subgroup specificity. These findings indicate that chronic diseases, preventive services, functional limitations, and socioeconomic factors jointly constitute an important explanatory basis for semantic stratification within the adult population.

#### 3.2.5 Public health intervention priority and pathway analysis

Finally, to further enhance the public health applicability of semantic subgroup analysis, this section examines intervention priorities and potential intervention pathways for different semantic subgroups within the adult population by considering the relationships among subgroup size, composite public health burden, cognitive reference burden, and dominant intervention dimensions. This analysis aims to further translate the preceding results of representation learning, semantic profiling, and cognitive reference validation into population-level identification and intervention clues from a public health perspective, rather than interpreting them as individual-level clinical diagnoses or deterministic risk predictions.

Figure 8 presents the differences among semantic subgroups in public health intervention priorities and potential intervention pathways. Figure 8A positions each subgroup along two dimensions, namely public health burden and cognitive-related burden, with bubble size representing subgroup size. The results show that although the low-burden subgroup accounts for a relatively large proportion of the population, it has a lower cognitive-related burden, whereas the preventive service gap subgroup, general health vulnerability subgroup, and health behavior risk subgroup show greater intervention relevance across different burden dimensions. Figure 8B further links each semantic subgroup to its main associated public health intervention dimensions, indicating that the corresponding intervention pathways differ across subgroups. For example, the general health vulnerability subgroup is more concentrated in dimensions related to general health and functional limitation, the health behavior risk subgroup is more closely associated with chronic disease and behavior-related risk dimensions, and the preventive service gap subgroup is connected to multiple dimensions, including socioeconomic vulnerability, functional limitation, and health behavior risk. Figure 8C summarizes the public health dimensions that should be prioritized for each subgroup in the form of targeted intervention signatures, further demonstrating that the proposed analytical framework can not only identify latent semantic subgroups within the adult population, but also provide structured evidence for public health resource allocation, priority population screening, and stratified intervention design. These targeted intervention signatures are intended to support population-level risk stratification and the identification of public health intervention priorities rather than to guide individualized clinical intervention or treatment decisions.

**Figure 8:**
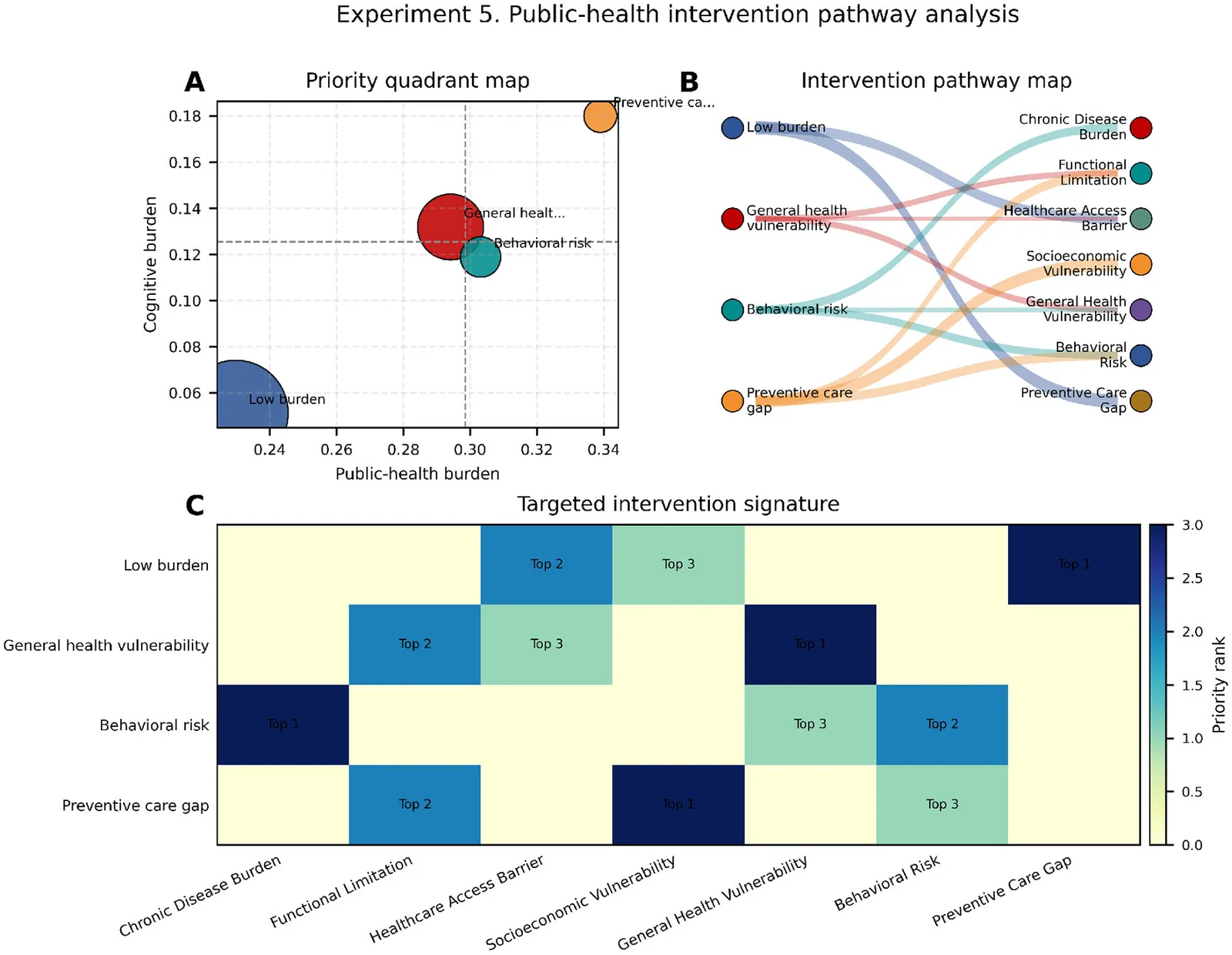
Public-health intervention pathway analysis. **(A)** Priority quadrant map of the identified subgroups. **(B)** Intervention pathways linking subgroups to public-health domains. **(C)** Targeted intervention priorities across subgroups.

## 4 Discussion

This study demonstrates that combining unsupervised deep representation learning with semantic profiling can identify interpretable latent subgroups from routine public health survey data and reveal differences in held-out cognitive reference variables. Unlike individual-level diagnosis or risk prediction, the present study focuses on population heterogeneity embedded in chronic disease burden, functional limitation, healthcare access, socioeconomic vulnerability, general health, health behaviors, and preventive service gaps. The learned representation space produced distinct subgroup profiles across public health dimensions, cognitive reference variables, key features, and intervention priorities, supporting its exploratory use for population stratification and public health resource planning.

The identified subgroups suggest that cognitive health-related vulnerability may accumulate through different public health pathways. The general health vulnerability subgroup was characterized mainly by poor general health, chronic disease, and functional limitation; the health behavior risk subgroup combined behavioral and chronic disease burdens; and the preventive service gap subgroup showed greater preventive-care and socioeconomic vulnerability. Although the low-burden subgroup had the lowest overall burden, “low burden” did not indicate the absence of risk. Its relatively elevated Preventive Care Gap score specifically reflected lower reported uptake of influenza and pneumococcal vaccination. Because healthcare access was represented separately, the present cross-sectional and self-reported data cannot determine whether this pattern was associated with reduced access, under-detection, or under-evaluation of other risks.

Public health strategies should therefore consider health services, chronic disease management, functional status, socioeconomic conditions, behaviors, and preventive care jointly. However, psychosocial coverage was limited. The 35 clustering variables included self-reported depressive disorder and several social or socioeconomic characteristics, but did not directly measure anxiety, loneliness, social isolation, or social support. These factors are important in multidomain dementia prevention and cognitive and cardiovascular health assessment (14–16). Accordingly, the targeted intervention signatures should be interpreted as population-level risk-stratification and resource-prioritization patterns rather than individualized clinical recommendations. In addition, because the Cognitive Decline module was limited to respondents aged at least 45 years in selected state–questionnaire-version strata, module availability, skip patterns, and missingness may influence the observed cognitive-reference patterns, which are not nationally representative prevalence estimates.

Repeated analyses across five independent random seeds supported the internal stability of the four-cluster solution, but population-split, state-level, cross-year, and independent external validation were not performed. Differences in population composition, variable definitions, questionnaire versions, and optional-module availability may therefore affect generalizability. Future studies should incorporate survey weighting, population-split and geographic validation, harmonized multi-year BRFSS data, longitudinal follow-up, and independent datasets, together with broader psychosocial, preventive-service, healthcare-utilization, and clinical diagnostic indicators.

## 5 Conclusion

Based on the BRFSS 2024 adult public health survey data, this study constructs an exploratory analytical framework that integrates unsupervised deep representation learning, semantic subgroup identification, public health profiling, and cognitive reference variable validation to reveal the latent distribution structure of cognitive health-related vulnerability embedded in routine public health variables. The results indicate that cognitive health-related problems in the adult population should not be simply understood as isolated memory or cognitive phenomena, but are jointly associated with multidimensional public health factors, including chronic disease burden, functional limitation, barriers to healthcare access, socioeconomic vulnerability, general health status, health behavior risk, and preventive service gaps. The public health significance of this framework lies in its ability to identify interpretable latent population structures from routine survey data without relying on clinical diagnostic labels, thereby providing data-driven evidence for priority population identification, public health resource allocation, preventive service optimization, and stratified intervention design. It should be noted that the findings of this study do not represent dementia diagnosis, disease prognosis assessment, or causal inference; future research should further incorporate sampling weights, alternative modeling approaches, cross-year data, and longitudinal or external data sources to examine the stability, representativeness, and generalizability of the identified subgroup structure.

## Data Availability

The original contributions presented in the study are included in the article/(supplementary material, further inquiries can be directed to the corresponding author.
