# Supplementary material for: Unsupervised discovery of public health subgroups associated with cognitive health vulnerability in adults: an analysis of BRFSS 2024
Source: Front Public Health. 2026 Sep 14;14:1878119. doi: 10.3389/fpubh.2026.1878119 (PMC13616714; doi:10.3389/fpubh.2026.1878119)
Supplement: Supplementary file 1 [file Table_1.docx]

**Appendix**

The 35 common public health features used for representation learning, their assigned public health domains or analytical roles, and the corresponding risk-direction coding.

| **Variable** | **Description** | **Assigned domain or analytical role** | **Risk-direction coding** |
| --- | --- | --- | --- |
| SEXVAR | Sex | Representation learning only; not included in domain burden calculation | Not direction-aligned for domain analysis; retained for autoencoder representation learning and standardized with the other input features. |
| GENHLTH | General health status | General Health Vulnerability | Min–max normalized; a higher original response category corresponds to a higher burden value. |
| PRIMINS2 | Health insurance coverage | Healthcare Access Barrier | Having insurance was coded as 0, whereas having no insurance was coded as 1. |
| PERSDOC3 | Personal doctor or healthcare provider status | Healthcare Access Barrier | Min–max normalized; a higher response category corresponds to a higher healthcare-access burden. |
| MEDCOST1 | Inability to obtain medical care because of cost | Healthcare Access Barrier | A reported cost-related barrier was coded as 1, whereas no cost-related barrier was coded as 0. |
| CHECKUP1 | Time since the last routine health checkup | Healthcare Access Barrier | Min–max normalized; a longer interval since the last routine health checkup corresponds to a higher burden value. |
| EXERANY2 | Participation in physical activity or exercise | Behavioral Risk | Participation in physical activity was coded as 0, whereas no reported physical activity was coded as 1. |
| CVDINFR4 | History of myocardial infarction | Chronic Disease Burden | A reported history of the condition was coded as 1, whereas no history was coded as 0. |
| CVDCRHD4 | History of coronary heart disease or angina | Chronic Disease Burden | A reported history of the condition was coded as 1, whereas no history was coded as 0. |
| CVDSTRK3 | History of stroke | Chronic Disease Burden | A reported history of the condition was coded as 1, whereas no history was coded as 0. |
| ASTHMA3 | History of asthma | Chronic Disease Burden | A reported history of the condition was coded as 1, whereas no history was coded as 0. |
| CHCSCNC1 | History of skin cancer | Chronic Disease Burden | A reported history of the condition was coded as 1, whereas no history was coded as 0. |
| CHCOCNC1 | History of cancer other than skin cancer | Chronic Disease Burden | A reported history of the condition was coded as 1, whereas no history was coded as 0. |
| CHCCOPD3 | History of chronic obstructive pulmonary disease, emphysema, or chronic bronchitis | Chronic Disease Burden | A reported history of the condition was coded as 1, whereas no history was coded as 0. |
| ADDEPEV3 | History of depressive disorder | General Health Vulnerability | A reported history of depressive disorder was coded as 1, whereas no history was coded as 0. |
| CHCKDNY2 | History of kidney disease | Chronic Disease Burden | A reported history of the condition was coded as 1, whereas no history was coded as 0. |
| HAVARTH4 | History of arthritis | Chronic Disease Burden | A reported history of the condition was coded as 1, whereas no history was coded as 0. |
| DIABETE4 | History of diabetes | Chronic Disease Burden | A reported history of the condition was coded as 1, whereas no history was coded as 0. |
| MARITAL | Marital status | Representation learning only; not included in domain burden calculation | Not direction-aligned for domain analysis; retained for autoencoder representation learning and standardized with the other input features. |
| EDUCA | Educational attainment | Socioeconomic Vulnerability | Reverse min–max normalized; lower educational attainment corresponds to a higher vulnerability value. |
| RENTHOM1 | Home ownership or rental status | Socioeconomic Vulnerability | Min–max normalized according to the original response coding; higher response categories correspond to higher vulnerability values. |
| VETERAN3 | Veteran status | Representation learning only; not included in domain burden calculation | Not direction-aligned for domain analysis; retained for autoencoder representation learning and standardized with the other input features. |
| EMPLOY1 | Employment status | Socioeconomic Vulnerability | Min–max normalized according to the original response coding; higher response categories correspond to higher vulnerability values. |
| INCOME3 | Annual household income | Socioeconomic Vulnerability | Reverse min–max normalized; lower household income corresponds to a higher vulnerability value. |
| DEAF | Deafness or serious hearing difficulty | Functional Limitation | The presence of the functional difficulty was coded as 1, whereas its absence was coded as 0. |
| BLIND | Blindness or serious vision difficulty | Functional Limitation | The presence of the functional difficulty was coded as 1, whereas its absence was coded as 0. |
| POORHLTH | Number of days poor physical or mental health limited usual activities | Functional Limitation | The reported number of days during the past 30 days in which poor physical or mental health limited usual activities was min–max normalized, with a greater number of days corresponding to a higher functional-limitation burden. |
| DIFFWALK | Difficulty walking or climbing stairs | Functional Limitation | The presence of the functional difficulty was coded as 1, whereas its absence was coded as 0. |
| DIFFDRES | Difficulty dressing or bathing | Functional Limitation | The presence of the functional difficulty was coded as 1, whereas its absence was coded as 0. |
| DIFFALON | Difficulty completing errands alone | Functional Limitation | The presence of the functional difficulty was coded as 1, whereas its absence was coded as 0. |
| SMOKE100 | History of smoking at least 100 cigarettes | Behavioral Risk | A reported smoking history was coded as 1, whereas no such history was coded as 0. |
| FLUSHOT7 | Receipt of influenza vaccination | Preventive Care Gap | Receipt of influenza vaccination was coded as 0, whereas non-receipt was coded as 1. |
| PNEUVAC4 | Receipt of pneumococcal vaccination | Preventive Care Gap | Receipt of pneumococcal vaccination was coded as 0, whereas non-receipt was coded as 1. |
| WTKG3 | Body weight | Representation learning only; not included in domain burden calculation | Not direction-aligned for domain analysis; retained as a continuous autoencoder input and standardized with the other input features. |
| HTM4 | Body height | Representation learning only; not included in domain burden calculation | Not direction-aligned for domain analysis; retained as a continuous autoencoder input and standardized with the other input features. |

Summary of age, Cognitive Decline module availability, state and questionnaire-version composition, and cognitive-variable response availability across the four semantic subgroups.

| **Semantic subgroup** | **Total sample, N** | **Age, mean ± SD** | **Age ≥45 years, n (%)** | **Module-available sample, n (%)** | **State and questionnaire-version distribution** | **Cognitive-variable valid/non-valid response rates (%)** |
| --- | --- | --- | --- | --- | --- | --- |
| Low burden | 295,286 | 50.48 ± 18.00 | 176,848 (59.9) | 50,200 (17.0) | All 19 states were represented; the three largest shares were Florida (10.3%), Maine (9.8%), and Indiana (9.7%). Questionnaire versions 10/11/12/20/21/22 accounted for 15.0/1.9/1.8/66.5/7.3/7.6%, respectively. | CIM: 84.2/15.8; WRY: 9.0/91.0; DIS: 9.1/90.9; HOU: 9.0/91.0; SOC: 9.0/91.0. |
| General health vulnerability | 108,820 | 61.21 ± 15.97 | 89,959 (82.7) | 26,486 (24.3) | All 19 states were represented; the three largest shares were Florida (12.0%), Maine (10.6%), and Indiana (9.5%). Questionnaire versions 10/11/12/20/21/22 accounted for 20.3/2.6/2.5/60.8/7.1/6.6%, respectively. | CIM: 86.1/13.9; WRY: 19.7/80.3; DIS: 19.8/80.2; HOU: 19.7/80.3; SOC: 19.5/80.5. |
| Behavioral risk | 34,256 | 68.76 ± 11.44 | 32,768 (95.7) | 9,673 (28.2) | All 19 states were represented; the three largest shares were Florida (11.4%), Maine (10.8%), and Indiana (10.3%). Questionnaire versions 10/11/12/20/21/22 accounted for 22.8/3.0/2.4/59.8/5.6/6.3%, respectively. | CIM: 86.1/13.9; WRY: 18.8/81.2; DIS: 18.9/81.1; HOU: 18.8/81.2; SOC: 18.7/81.3. |
| Preventive care gap | 19,308 | 66.69 ± 12.71 | 17,989 (93.2) | 5,445 (28.2) | All 19 states were represented; the three largest shares were Florida (12.2%), Indiana (11.1%), and Maine (9.2%). Questionnaire versions 10/11/12/20/21/22 accounted for 23.2/2.6/2.5/60.4/5.7/5.6%, respectively. | CIM: 84.5/15.5; WRY: 24.9/75.1; DIS: 25.1/74.9; HOU: 25.0/75.0; SOC: 24.7/75.3. |

*Note: The module-available sample was operationally defined as respondents aged at least 45 years who belonged to a state–questionnaire-version stratum in which CIMEMLO1 was observed. The module-available percentage was calculated relative to the complete sample of the corresponding subgroup. State percentages and questionnaire-version percentages were calculated within the module-available sample of each subgroup. For conciseness, only the three states with the largest shares are shown; all 19 participating states were represented in each subgroup. Cognitive response availability is presented as valid/non-valid percentages among module-available respondents. Valid responses were codes 1 or 2, whereas non-valid responses included questionnaire blanks or skip-pattern omissions, “do not know/not sure,” refusals, and other non-valid responses. CIM = CIMEMLO1; WRY = CDWORRY; DIS = CDDISCU1; HOU = CDHOUS1; SOC = CDSOCIA1.*
